# Whole-genome sequence of an ammonia-nitrogen-degrading *Stutzerimonas kunmingensis* strain JD1-4

**DOI:** 10.1128/mra.00582-26

**Published:** 2026-10-27

**Authors:** Jinyang Zhang, Xin Liao, Yeshi Yin, Huahai Chen

**Affiliations:** 1 Guangxi Academy of Marine Sciences, Guangxi Mangrove Research Center, Guangxi Key Lab of Mangrove Conservation and Utilization 425048, Beihai, China; 2 College of Animal Science and Technology, Guangxi University 12664, Nanning, Guangxi, China; 3 Guangxi Key Laboratory of Animal Reproduction, Breeding and Disease Control, Nanning, Guangxi, China; 4 Guangxi Academy of Marine Sciences, Guangxi Academy of Sciences 245477, Nanning, Guangxi, China; California State University San Marcos, San Marcos, California, USA

**Keywords:** whole-genome sequence, ammonia nitrogen degrading, *Stutzerimonas kunmingensis*

## Abstract

The genome of the ammonia-degrading bacterium *Stutzerimonas kunmingensis* JD1-4, isolated from mangrove sediment in Guangxi, was sequenced using PacBio Sequel II and Illumina NovaSeq 6000 platforms, yielding a single 4,852,889 bp chromosome without plasmids. Annotation identified 42 nitrogen metabolism genes, providing the genetic basis for its ammonia-degrading capability.

## ANNOUNCEMENT

Ammonia nitrogen pollution is a major concern in marine environments, driving the need for microbial resources capable of efficient nitrogen removal (1, 2). Members of the family Pseudomonadaceae are known for their metabolic versatility and are frequently implicated in nitrogen cycling (3, 4). Here, an ammonia-nitrogen-degrading bacterium, *Stutzerimonas kunmingensis* JD1-4, was isolated from intertidal mangrove sediment (10–15 cm depth) in Beihai, Guangxi, China (21°28′ N, 109°43′ E), in October 2024. Isolation was performed by serial dilution and plating on Gauze’s medium under anaerobic conditions at 30 °C for 48 h. The strain removed 94.57% of 200 mg/L NH₄^+^-N within 48 h in heterotrophic nitrification medium containing (NH_4_)_2_SO_4_ and CH_3_COONa at 37 °C, 160 rpm, measured by Nessler’s reagent spectrophotometry (5).

For whole-genome sequencing of strain JD1-4, genomic DNA was extracted using the EZ-10 Spin Column Bacterial Genomic DNA Isolation Kit (Sangon Biotech, Shanghai, China). Sequencing libraries were prepared with the NEBNext Ultra II DNA Library Prep Kit (New England Biolabs) and the SMRTbell Express Template Prep Kit 2.0 (Pacific Biosciences). Sequencing generated 8,370,400 Illumina reads (2 × 150 bp, 1.26 Gb) and 95,852 PacBio reads (N50 = 17,382 bp, 850.55 Mb). After quality filtering and error correction, hybrid assembly with Unicycler v0.5.0 (6) yielded a single circular chromosome of 4,852,889 bp (62.45% G + C) with no plasmids; circularity was confirmed by terminal repeat overlap. BUSCO v5.4.7 analysis indicated 98.7% genome completeness (7). Average nucleotide identity (ANI) was calculated using gcPathogen v1.0 (8), accessed on 20 April 2026. The highest ANI value (96.98%) was obtained with *S. kunmingensis* GCA_900114065.1.

PGAP v6.10 (9) identified 4,424 protein-coding genes, and KEGG revealed 42 genes mapped to nitrogen metabolism, including *glnA*, *gdhA*, *narG*, *narH*, *narI*, *nirK*, and *norB*. No ammonia monooxygenase genes were detected, suggesting that ammonia removal proceeds primarily through assimilation and a heterotrophic nitrification-aerobic denitrification pathway, consistent with other members of the Pseudomonadaceae family (10). This genome will facilitate further investigations into the genetic mechanisms of marine ammonia degradation and the development of microbial agents.

## Data Availability

The whole-genome shotgun sequencing data have been deposited in GenBank under accession number JBWQFJ000000000. The associated BioProject and BioSample accession numbers are PRJNA1444553 and SAMN56760216, respectively. Raw sequencing reads are available in the Sequence Read Archive under accession number PRJNA1444556.
